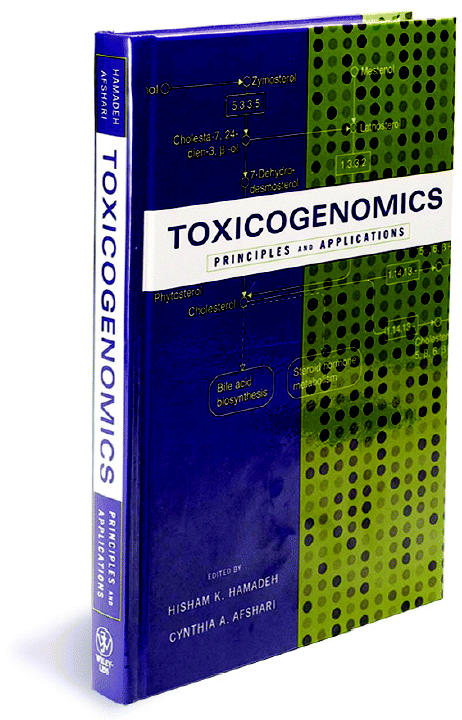# Toxicogenomics: Principles and Applications

**Published:** 2004-11

**Authors:** Gilbert S. Omenn

**Affiliations:** Gilbert S. Omenn is professor of internal medicine, human genetics, and public health at the University of Michigan. His research interests include cancer proteomics, chemoprevention of cancers, public health genetics, science-based risk analysis, and health policy. He leads the Plasma Proteome Project for the international Human Proteome Organization and is president-elect of the American Association for the Advancement of Science.

Edited by Hisham K. Hamadeh and Cynthia A. Afshari

Hoboken, NJ:Wiley-Liss, 2004. 361 pp. ISBN: 0-471-43417-5. $69.95 cloth

This timely book presents basic toxicology for molecular biologists and detailed approaches and methods of gene expression, protein, and metabolic global analyses for toxicologists and others learning to use such methods. There are high expectations that “molecular signatures” will become useful as biomarkers of exposure, early effect, and differential susceptibility and reveal targets for new drugs. I particularly appreciated the effort to link genotypes to phenotypes in the form of “target organ toxicity patterns”—in liver, kidney, lung, nervous system, skin, reproductive system—-from Chapter 1 onward. The detailed pathology and physiology along the periportal–centrilobular gradient in liver, combined with references to mRNA analyses of 15 hepatotoxins, and along the segments of the renal tubule exposed to cisplatin or ochratoxins, illustrate (e.g., Chapter 8) the opportunity to link traditional toxicology and pathology with the molecular analyses. Conversely, global molecular analyses have begun to reveal many previously unsuspected or unknown targets for desired and adverse effects of drugs and other chemicals.

The aim of the new approaches is to transform toxicology from descriptive to predictive, including prediction of *in vivo* effects from *in vitro* models and other species. Technical features are presented in considerable detail. Innovative manufacture, miniaturization, scanning, and statistical analyses of DNA microarrays have yielded sequence and gene expression information with remarkable throughput. Many technical advances are still needed, such as better evidence that fluorophores match up well in two-color experiments. Statistical analyses of a methapyrilene study vividly demonstrate sources of “false discoveries” (Chapter 6). Differential cell loss in heterogeneous tissues will change mRNA ratios, requiring estimates by pathologists of changes in tissue composition. Expressed sequence tags unannotated for function may be discriminants for disease associations (Chapter 9). *In vitro* response patterns may not match *in vivo* patterns (Chapter 10). When pathways and regulation are highly conserved, yeast cells or other model organisms may reveal a lot about the multiple targets and interactions of a drug or its intended protein target.

“Toxicogenomics” is defined explicitly to embrace proteins and metabolites as the key effector classes in functional genomics. There are two chapters on proteomics and one on metabolomics and metabo-nomics. Multiple, rapidly evolving fractionation, chemical tagging, mass spectrometry, microarray format affinity methods, and database search algorithms are highlighted; key challenges are much higher throughput, validation of protein identifications, and quantitation. Plasma and tissue lysate proteomes are very complex mixtures, reflecting the huge range of concentrations and many isoforms of large numbers of proteins, and the dynamic nature of the structure–function relationships. International cooperative strategies are recommended (Chapter 12). Proteins are targets of many environmental agents, with potential effects on all functions and pathways in the cell, including the posttranslational modification of proteins themselves. Peptide adducts with electrophiles have been characterized and mapped on target proteins with tandem mass spectrometry scans and SALSA software (Chapter 13); finding low abundance targets and focusing instrument time on peptides of highest interest are big challenges. Finally, metabolic profiles of toxicologic exposures have yielded many potential markers of early effects.

The editors, formerly at the National Institute of Environmental Health Sciences (NIEHS) Center for Toxicogenomics and now at Amgen Inc., and their colleagues from the NIEHS, academe, and many companies have highlighted emerging techniques, practical laboratory planning, and explicit biostatistical and bioinformatic interpretations. The book is well referenced, with particular attention to web-based resources. The emphasis on methods rather than signatures reveals the still-early nature of this promising field.

## Figures and Tables

**Figure:**